# The effectiveness and safety of recombinant human growth hormone combined with alginate dressing in the treatment of diabetic foot ulcer: A protocol for systematic review and meta-analysis

**DOI:** 10.1097/MD.0000000000023984

**Published:** 2021-02-05

**Authors:** Deng-Rong Zhou, Hai-Yan Deng, Lin-Li Pu, Shao-Lan Lin, Rong Gou, Feng-Ling Wang

**Affiliations:** a Department of Nursing; b Department of Endocrinology; c Department of Encephalopathy, Haikou Hospital of Traditional Chinese Medicine, No. 45 Jinpan Road, Longhua District, Haikou, Hainan Province, 570216, China.

**Keywords:** alginate dressing, diabetes complications, diabetic foot ulcer, randomized controlled trial, recombinant human growth hormone, system review and meta-analysis

## Abstract

**Background:**

Diabetic foot ulcer (DFU) is one of the serious complications of diabetes. It is the result of a joint effect of lower extremities vascular lesions, neuropathy, and infection, which require amputation and even threaten the life of the patient. At present, the conventional treatment for DFU includes infection control, wound care, wound reduction, reduction of foot pressure, use of dressings that are beneficial to wound surface healing, etc, but the effectiveness is not satisfactory. Recombinant human growth hormone and alginate dressing have been used in clinical, but there is lack of the relevant evidence of its effectiveness and safety, so this study evaluates the clinical effectiveness and safety of recombinant human growth hormone combined with alginate dressing in the treatment of DFU by systematic evaluation, the purpose is to provide a theoretical basis for the treatment of diabetic foot ulcer.

**Methods:**

This study mainly retrieves the randomized controlled trial of recombinant human growth hormone combined alginate dressing in the treatment of DFU in 7 electronic databases, such as PubMed, EMbase, Cochrane Library, SinoMed, CNKI, WANGFANG database, and VIP database. All the retrieval dates of database are from the establishment of the database until May 31, 2020. At the same time, searching the related degree papers, conference papers, and other gray literature by manual. The original literature data are independently screened and extracted by 2 researchers on the basis of inclusion and exclusion criteria and literature information sheets, and cross-checked and resolved through group discussions and consultations when there are differences of the opinion. Assessing the methodological quality of inclusion in the study based on the “Bias Risk Assessment Form” of the Cochrane Collaboration Network. Using the software of RevMan 5.3.3 and STATA 13.0 for statistical analysis.

**Results:**

This study compares the main and secondary outcome indicators by systematic evaluation and it will provide strong evidence of recombinant human growth hormone combined alginate dressing in the treatment of DFU.

**Ethics and dissemination:**

All data in this study are obtained through the web database and do not involve humans, so ethical approval is not suitable for this study.

**OSF registration number:**

DOI 10.17605/OSF.IO/W6P24.

**Conclusion:**

This study will give positive conclusions about the effectiveness and safety of recombinant human growth hormone combined alginate dressing in the treatment of DFU.

## 1 Introduction

Diabetes is a group of metabolic diseases characterized by high blood sugar and it causes by insulin secretion or deficiency of action, or both. Diabetes is becoming a public health problem as the improvement of people's living standards and the changes of their lifestyles.^[1]^ Globally, the number of people with diabetes is reported to have reached 382 million in 2013, and that number will exceed 592 million by 2035. With a prevalence rate of 9.7%, China has become the fastest growing region of diabetes in the world and the world's largest diabetes country.^[2,3]^ The harm of diabetes is mainly reflected in complications, it has reported that the risk of cardiovascular complications in diabetic patients is 2 to 4 times higher than that in normal people, and diabetes is the leading cause of non-traumatic amputation, blindness, and advanced kidney disease.^[4,5]^ As one of the serious complications of diabetes, diabetic foot ulcer (DFU), its high incidence, high hospitalization rate and high disability rate seriously threaten the quality of life of diabetic patients. According to statistics, diabetic patients have a 12% to 25% chance of developing DFU, and the recurrence rate within 5 years after ulcer healing is 50% to 70%.^[6]^ About a quarter of people hospitalized with diabetes are associated with DFU complications, and there is 1 amputation due to DFU every 30 seconds in the world has a 50% increased risk of losing another leg after 3 years, the leading cause of non-trauma amputation, with a death rate of 13% to 17%.^[7,8]^ DFU not only affects the quality of life of diabetic patients, but also brings a heavy burden to their families and society. In the United States, the total cost of treating a final amputation due to a severe DFU infection is already as high as $190,000; in China, the cost of treating non-severe DFU is $1673; in India, a patient's total income of 5.7 years can only treat a foot ulcer surface.^[9]^ It can be seen that DFU has become a public challenge, the development and prognosis of foot ulcer is the result of multi-factor interaction, and it is closely related to neurological dysfunction, exovascular lesions, and infection. It is necessary to study the causes of foot ulcer healing and perfect its treatment plan to explore effective early prevention and treatment methods.^[10]^

The clinical treatment of DFU is difficult, long course of illness, high cost of treatment, poor quality of life, so surgical treatment has become the main method of diabetic foot ulcer, but how to improve the quality of preoperative surface foundation, reduce preoperative preparation time, improve the rate of wound healing after surgery has been listed as the common problem need to be solved urgently by the world's major health organizations.^[11]^ Growth hormone (GH) is a protein hormone secreted by the pituitary frontal leaves, controlled and regulated by the hypothalamus, which consists of 191 amino acid residues, the relative component is about 2.2 × 104, and it contains 2 pairs of disulfide bonds, no glycosylation, with a high degree of species specificity. GH is regulated by the GH secretion receptor in the human body, the main biological function is directly involved in the body metabolism and indirectly promote the growth of the body.^[12]^ Recombinant human growth hormone (r-hGH) is produced by recombinant gene technology and its chemical composition includes amino acid sequence and amino acid composition determined to be identical to pituitary growth hormone. In the 1980s, r-hGH was made and officially listed through gene recombination technology, which initially mainly treated patients with growth hormone deficiency (GHD), and it was then widely used in the fields of pituitary degeneration, bone and cartilage repair, burns, surface repair, plastic surgery, and so on. After long-term clinical research and practice observation, a better therapeutic effect has been obtained.^[13]^ In recent years, with the further study of GH, its application in the field of DFU repair has gradually increased.^[14,15]^ Dressing is widely used in wound care, protecting wounds and promoting wound healing, and it is a key material for treating DFU, of which alginate dressing is the most representative. Alginate dressing has high water absorption, exists in the form of calcium sea alginate or sodium sea alginate, and it can be combined with collagen. Alginate comes into contact with the surface of the wound to form a gel that can be removed with the dressing or rinsed with sterile saline. Alginate dressing contains calcium alginate or sodium sea alginate is highly hydrophilic and can absorb large amounts of wound oozing, so it is commonly used to treat very wet wounds, absorb excess water, and avoid skin impregnation.^[16]^ International guidelines for DFU treatment suggest that DFU should choose a dressing that keeps wounds moist, so alginate dressing has gradually been used in the treatment of DFU in recent years.^[17]^

At present, recombinant human growth hormone and alginate dressing have been used in clinical treatment of diabetic foot ulcer, but due to the lack of high quality, multi-level, and multi-angle clinical trial supporting, so the effectiveness and safety of recombinant human growth hormone and alginate dressing in DFU patients is still controversial. This study evaluates the randomized controlled trials of recombinant human growth hormone combined alginate dressing in the treatment of DFU by systematic evaluation.

## 2 Study objective

The purpose of this study is to find evidence-based medical evidence for the effectiveness and safety of recombinant human growth hormone combined alginate dressing in the treatment of DFU, so as to provide new clinical ideas and theoretical basis for the diagnosis and treatment of DFU in the future.

## 3 Methods

### 3.1 Study registration

OSF registration number: DOI 10.17605/OSF.IO/W6P24 (https://osf.io/w6p24).

### 3.2 Document inclusion criteria

#### 3.2.1 Inclusion criteria

1. All cases meet the WHO Diagnostic Criteria for Type 2 Diabetes in 1999.^[18]^
2. Meet the diagnosis criteria of diabetes foot.
3. The ulcer area ≤6 cm^2^.
4. During the hospitalization, the patient's condition is stable, the blood sugar is stable, and diabetic foot ulcer is the main clinical manifestations.
5. Wagner is rated 2 to 3.
6. The blood vessels in the lower extremities function well, and the ankle brachial index (ABI) ≥ 0.9.

#### 3.2.2 Type of studies

Collecting the randomized controlled trials published in Chinese or English on recombinant human growth hormone combined with alginate dressing in the treatment of DFU.

#### 3.2.3 Type of participants

Patients with type I or type II diabetes and open DFU wounds. There are no restrictions on the cause of DFU in this study, and randomized controlled trials in DFU patients with neurotic or ischemia or ischemia are eligible for inclusion.

#### 3.2.4 Intervention

Patients in the control group were treated with alginate dressing for external use and basic treatment of diabetes.

Patients in the experimental group were treated with recombinant human growth hormone + alginate dressing for external use + basic treatment of diabetes. The specifications, dosing, and treatment time of the drug are unlimited.

### 3.3 Document exclusion criteria

1. Persons with severe cognitive impairment and non-cooperation.
2. Persons with apparent necrosis and osteomyelitis.
3. Patients with malignant tumors.
4. Persons suffering from cellular tissue inflammation who have not been treated.
5. Drugs that interfere with this study have been used in the last 1 month.
6. Exclusion of diabetics who are pregnant.
7. Patients using drugs that affect wound healing: for example, glucocorticoids, chemotherapy, anticoagulants, immunosuppressants, etc.
8. Literature with incomplete reviews, animal studies, and data reports.

### 3.4 Type of outcome measures

#### 3.4.1 Main outcome indicators

1. Total effective rate: it is evaluated by healing, effectiveness, and ineffectiveness. Healing: ulcer or gangrene have fully healed, or scarred. Effectiveness: ulcer or gangrene surface reduction, significant reduction of local secretions, gangrene shows a large area of shedding state, and some new granulated tissue. Ineffectiveness: ulcer or gangrene invasive surface aside, secretions does not significantly reduce, or even increase. Total effective rate = healing + effectiveness/total number of cases × 100%.
2. Local symptom scores: the total score is 36 points, the higher the score, the more serious local symptoms.
  (1) Wound surface depth: Healing (0 points), <0.5 cm (2 points), 0.5 to 1 cm (4 points), >1 cm (6 points).
  (2) The coverage rate of rotten meat: none (0 points), <30% (2 points), 30% to 70% (4 points), >70% (6 points).
  (3) The range of red and swollen: none (0 points), from the edge of the wound <1 cm (2 points), from the edge of the wound 1 to 3 cm (4 points), from the edge of the wound >3 cm (6 points).
  (4) Meat buds: healing (0 points), red shoots, small particles (2 points), granules light red or slightly red (4 points), granules edema, meat color is not fresh (6 points).
  (5) Secretions: none (0 points), serum-like secretions (2 points), pus thick (4 points), pus thick, skunk (6 points).
  (6) Pain level: none (0 points), occasional or sustained minor pain (2 points), pain is tolerable, does not affect normal life (4 points), pain is unbearable, need to use analgesics (6 points).

#### 3.4.2 Additional outcome indicators

1. Occurrence of adverse reactions: including bleeding, edema, infection, dermatitis, etc.
2. Coagulation function: active partial clotting enzyme time and fibrinogen content.
3. High value of ankle brachial index, ABI, high value of systolic pressure of the ankle artery (back artery or back artery)/high value of systolic pressure of the artery.
4. The healing time of wound surface.
5. Cumulative number of drug changes.

### 3.5 Search strategy

The computer fully retrieves PubMed, EMBASE, Cochrane Library, CNKI, SinoMed, WANFANG Database, VIP Database, and the retrieval time range is from the establishment of the database until May 31, 2020. Collecting the randomized controlled trials of recombinant human growth hormone combined alginate dressing in the treatment of DFU, the search method is to combine the subject word with the free word, and try to search for synonyms, and determine the search strategy after many pre-retrievals, according to the specific situation of strategic, different databases will be adjusted. In addition, the references to all relevant articles are screened to ensure full documentation. Results of literature search using PubMed database (Table 1).

**Table 1:** Results of literature search using PubMed database.

Database name	Search strategy
PubMed	1. Human Growth Hormone [MeSH]
	2. (Recombinant Human Growth Hormone[Ti/Ab]) OR (Growth Hormone, Human[Ti/Ab]) OR (hGH (Human Growth Hormone)[Ti/Ab]) OR (Somatropin (Human)[Ti/Ab]) OR (Somatotropin (Human)[Ti/Ab]) OR (Somatropin[Ti/Ab]) OR (Serostim[Ti/Ab]) OR (Zomacton[Ti/Ab]) OR (Cryo-Tropin[Ti/Ab]) OR (Cryo Tropin[Ti/Ab]) OR (CryoTropin[Ti/Ab]) OR (Recombinant Human Growth Hormone (Mammalian)[Ti/Ab]) OR (r-hGH-M[Ti/Ab]) OR (r-hGH(m)[Ti/Ab]) OR (Humatrope[Ti/Ab]) OR (Umatrope[Ti/Ab]) OR (Maxomat[Ti/Ab]) OR (Norditropin[Ti/Ab]) OR (Norditropin Simplexx[Ti/Ab]) OR (Norditropine[Ti/Ab]) OR (Nutropin[Ti/Ab]) OR (Omnitrope[Ti/Ab]) OR (Saizen[Ti/Ab]) OR (Genotropin[Ti/Ab]) OR (Genotonorm[Ti/Ab])
	3. Alginate[MeSH]
	4. (Alginate[Ti/Ab]) OR (Kaltostat[Ti/Ab]) OR (Vocoloid[Ti/Ab]) OR (Calginat[Ti/Ab]) OR (Potassium Alginate[Ti/Ab]) OR (Alginate, Potassium[Ti/Ab]) OR (Alginic Acid, Potassium Salt[Ti/Ab]) OR (Sodium Alginate[Ti/Ab]) OR (Alginate, Sodium[Ti/Ab]) OR (Alginic Acid, Sodium Salt[Ti/Ab]) OR (Alginic Acid, Sodium Salt[Ti/Ab]) OR (Kalrostat 2[Ti/Ab]) OR (Sodium Calcium Alginate[Ti/Ab]) OR (Alginate, Sodium Calcium[Ti/Ab]) OR (Calcium Alginate, Sodium[Ti/Ab]) OR (Barium Alginate[Ti/Ab]) OR (Alginate, Barium[Ti/Ab]) OR (Alginic Acid, Barium Salt[Ti/Ab]) OR (Calcium Alginate[Ti/Ab]) OR (Alginate, Calcium[Ti/Ab]) OR (Alginic Acid, Calcium Salt[Ti/Ab]) OR (Copper Alginate[Ti/Ab]) OR (Alginate, Copper[Ti/Ab]) OR (Alginic Acid, Copper Salt[Ti/Ab]) OR (Alloid G[Ti/Ab]) OR (Kalrostat[Ti/Ab]) OR (Xantalgin[Ti/Ab])
	5. Dressing[Ti/Ab]
	6. Diabetic Foot[MeSH]
	7. (Foot, Diabetic[Ti/Ab]) OR (Diabetic Feet[Ti/Ab]) OR (Feet, Diabetic[Ti/Ab]) OR (Foot Ulcer, Diabetic[Ti/Ab])
	8. 1 OR 2
	9. 3 OR 4
	10. 9 AND 5
	11. 6 OR 7
	12. 8 AND 10 AND 11

### 3.6 Study selection and data extraction

#### 3.6.1 Literature screening

**Figure 1:**
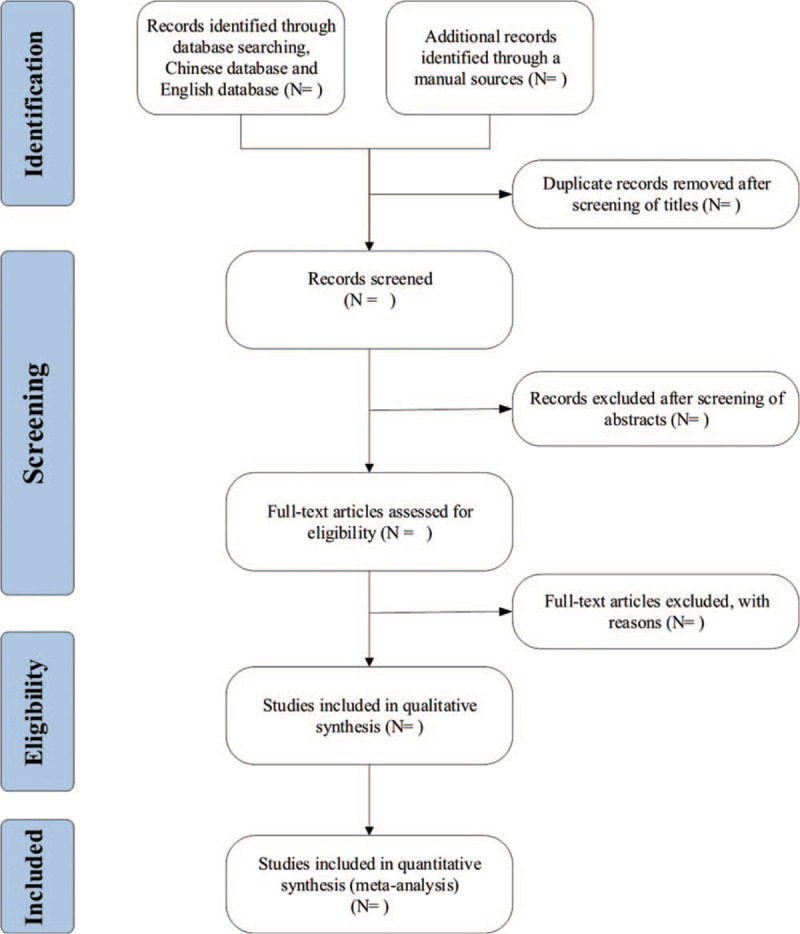
Flow diagram of the present meta-analysis.

1. The relevant literature is retrieved and a preliminary assessment is carried out independently by the 2 evaluators.
2. On the basis of inclusion and exclusion criteria, the 2 evaluators independently review and screen the literature, discuss or seek the views with the third evaluator in the event of disagreement, and document the reasons for excluding the literature.
3. Extracting the data by using the pre-designed extract tables and summarizing: 2 evaluators independently extract data and discuss differences resolution, and if there is a data deficiency in the included study, contacting the original author of the study to obtain the relevant information. Flow diagram of the present meta-analysis (Fig. 1).

#### 3.6.2 Data extraction

Extracting the following data from the data extraction table: author and publication time; the country of participant; DFU type; sample size randomly assigned to control group and experimental group; the basic information of patients (sample size, sex, age, course of disease); the details of treatment plan of control group and experimental group; the details of the same interventions in both groups of participants; the data on main and secondary outcome indicators: the sample size of counting data, the average and standard deviation of measurement data.

### 3.7 Assessment of risk of bias in included studies

The quality of included studies is evaluated by using the bias risk assessment tool recommended by the Cochrane System Reviewer Manual. A total of 7 evaluation entries, including:

(1) Random method.
(2) Allocation concealment.
(3) Implementation bias.
(4) Follow-up bias.
(5) Measurement bias.
(6) Reporting bias.
(7) Other bias.

Each evaluation entry has a “High,” “Unclear,” and “Low” 3 levels of bias risk. “High” refers to the failure to implement a group of allocation concealment, the intervention is not uniform, and the subjects withdraw from the trial research, random method is error, measurement results method is not uniform, blind method is not used, the outcome data is incomplete, or failure to report the main key outcomes that should be included, etc; “Unclear” means that the specific situation or incomplete information is not stated in the text; “Low” means that the random method describes it in detail and correctly, using the appropriate blind method, or whether or not the use of blind method does not affect the results of the trial, the outcome data is complete, or the missing data does not affect the outcome analysis, there are no other problems.

### 3.8 Statistical method

The RevMan 5.3.3 software provided by Cochrane Collaboration Network is used for meta-analysis. Heterogeneity tests are based on forest maps and *I*^2^ tests, the test level *α* is 0.05. The counting data is represented by odds ratio (OR) or relative risk (RR) and 95% CI, and the metering data is represented by a standardized mean difference (SMD) and 95% CI. The heterogeneity between the results of each study is tested by using Cochrane Q. When the studies with better homogeneity (*P* ≥ .1, *I*^2^ ≤ 50%), the fixed effect model is used, while the meta-analysis is carried out by using the random effect model for the studies with more obvious heterogeneity. In the forest map, the point estimate of each study effect is expressed in square, the weight of each study is represented by the square, the line extending on both sides of the square represents the confidence interval of the effect amount, the length of the line is inversely compared with the confidence interval of the effect amount, and the longer the segment, the wider the confidence interval, the less accurate the result is, and vice versa. When the 95% CI horizontal line of the study RR intersects the invalid vertical line (the scale of horizontal coordinate is 1), it is not statistically significant to indicate the difference between the occurrence rate of the experimental group and the control group, when the 95% horizontal line of the study RR falls on the right side of the invalid line, the occurrence rate of an event in the control group can be considered to be greater than the occurrence rate of an event in the experimental group, the experimental factor can increase the occurrence of an event, and it clinically considers the effectiveness of the experimental group to be better than the control group; otherwise, it is the opposite conclusion.

### 3.9 Sensitivity analysis

If there is obvious statistical heterogeneity between the included studies, it needs to conduct the analysis results for the sensitivity analysis.

(1) Excluding studies of lower quality, excluding studies with less stringent designs, excluding studies with large or small sample sizes, excluding unresolved studies, and then conducting meta-analysis again to observe whether the conclusions have changed.
(2) After changing the study type and selecting different effect models for the same data, it is observed whether the effect merge value and confidence interval have changed.

If the results of meta-analysis do not change significantly before and after the sensitivity analysis, indicating that the results of this meta-analysis are reliable, and if the results of meta-analysis change significantly before and after the sensitivity analysis, indicating that the reliability of the meta-analysis results is poor, it is important to clearly use heterogeneity sources, the results and conclusions need to be careful.

### 3.10 Publication bias

Using funnel plot to indicate publication bias, the horizontal coordinates of funnel plot to represent the effect size, ordinates represent the sample size by using the scatter plot. If the effect points of the included study on the axis of the collection is an inverted funnel shape, the left and right is basic symmetry, indicating there is no publication bias, and using Egger's test to detect the specific situation of publication bias. If *P* > .05, indicating that there is no publication bias, on the other hand, indicating that there is publication bias, which can be used to further clarify the symmetry of the funnel plot by linear regression, and to find out the intercept of regression equation and 95% CI, and to analyze the possible causes of publication bias.

### 3.11 Grading the quality of evidence

This study uses the method of Grading of Recommendations Assessment, Development, and Evaluation (GRADE) to evaluate the evidence quality and recommended strength of the study results, and it is divided into high (A), medium (B), low (C), and very low (D). The starting of RCT is high quality, and studying the bias risk, insistency, inexactness, indirectness, and publication bias that will be considered to downgrade and indicating the downgrade factors in the study results. The process of evaluation is conducted independently by the 2 researchers after the training, and discussing and resolving with the third researcher in case of disagreement.

## 4 Discussion

The mechanism of diabetic foot ulcer is complex, including the result of a combination of vascular lesions, neuropathy, and accompanying infection. The occurrence of DUF mainly includes 2 aspects:

(1) Vascular lesions are the main factors in the emergence of DFU, diabetes mellitus (DM) patients’ high blood sugar can aggravate the endothelial damage of blood vessels. In addition, its anti-agglutination effect is weakened, thus aggravating the formation and development of atherosclerosis. Atherosclerotic plaque is formed after atherosclerosis, so that it deposits a large amount of calcium in the patients’ blood vessels, thus affecting the formation of collateral circulation of blood vessels in the distal part of the body. DM patients are often accompanied by vascular spasms, which cause blood movement disorders at the end of the limb, most of which are associated with insulin resistance in DM patients.^[19]^ In addition, patients with DM disease have narrow blood vessels, rough blood vessel walls, increased fibrinogen and globulin, high blood viscosity and slow blood flow, which cause changes in the patient's hemodynamics. The change of hemodynamics can further increase the risk of foot ulcer, and patients often have no conscious symptoms until ulcers occur. The reduction of blood supply at the extremity of DFU patients hinders the wound healing, thus increasing the risk of foot lesions and infection.^[20]^
(2) Neuropathy and excessive mechanical force are the starting factors that cause ulcer in the lower extremities of DM patients. Neuropathy in DFU patients includes sensory neuropathy and mild peripheral neuropathy. Among them, sensory neuropathy makes patients lack of neuroprotective mechanism, resulting in sensory paralysis, and significantly reducing the patient's ability to protect themselves. Peripheral neuropathy causes the skin's blood regulation and control of perspiration to weaken, resulting in decreased elasticity of the foot tissue, thereby increasing the incidence rate of ulcer.^[21]^ In addition, the infection is the direct cause of amputation of DFU patients, due to peripheral neuropathy, the normal sweating ability of the sweat glands weakened, making it easier for bacteria to plant deep tissue. In addition, the inflammatory chemotaxis of DFU patients is weakened and the resistance is reduced. It is highly susceptible to cause infection and infection migration, which can seriously lead to amputation or even death.^[22]^

At present, the mechanism for recombinant human growth hormone in the treatment of DFU may have^[23]^:

(1) The effect of promoting cell proliferation and growth: the injection of growth hormone can stimulate the continuous proliferation of endoblast cells, endocrine cells, etc in wound tissue, thus accelerating the deposition of wound collagen, so that accelerates the re-epithelialization process of the ulcer wound and reduces the healing time of the wound.
(2) Immunomodulation effect: DFU patients are prone to cause infection and infection migration, may be due to the long-term high blood sugar of DFU patients, the tending effect of body inflammatory weakened, the decreased resistance. GH is a strong inducer of the immune system and a hormone that promotes the protein synthesis, which plays an important role in regulating the production and function of immune cells. It stimulates the synthesis of lymphocyte DNA, enhances the proliferation of macrophages and lymphocytes, and stimulates the production of immunoglobulins. In addition, growth hormone has been found in in-body experiments to improve the function of the immune system, which can promote the chemicalization effect, stimulate monocytes to enhance their phagocytic function, and improve the reactivity of T lymphocytes.
(3) Angiogenesis: Studies have confirmed that GH is an angiogenesis factor, which stimulates the division of endothelial cells and angiogenesis, and helps to promote the production of vascular growth factors and the expression of their function. Alginate dressing plays a role in wound healing to provide a moist environment for the wound, absorbing oozing, reducing pain in patients, reducing the infection risk in the wound, and helping the wound stop bleeding.^[24]^

It has the following advantages:

(1) It is easy to use, and it is safe. The dressing uses sterile sealed envelope. It can be used after opening the protective film. The soft gel formed by the dressing will not adhere to the fragile tissues of the wound bed and can be easily removed from the wound.
(2) Wetting wound healing environment, allowing gas exchange, which is the wound bed surface healthy growth of the necessary conditions, so as to prevent the formation of eschar and reduce the formation of scar.

At present, due to the lack of evidence-based medical evidence, the effectiveness and safety of recombinant human growth hormone and alginate dressings in DFU patients is debatable. In this study, the clinical effectiveness and safety of recombinant human growth hormone combined alginate dressing in the treatment of DFU are analyzed by systematic evaluation. Through this study, it will provide a positive evidence-based medical evidence to help the treatment of DFU and benefit more patients with DFU.

## Author contributions

**Conceptualization:** Deng-Rong Zhou, Feng-Ling Wang.

**Data curation:** Deng-Rong Zhou, Hai-Yan Deng, Lin-Li Pu.

**Formal analysis:** Deng-Rong Zhou, Hai-Yan Deng, Feng-Ling Wang.

**Funding acquisition:** Feng-Ling Wang.

**Methodology:** Deng-Rong Zhou, Lin-Li Pu, Shao-Lan Lin.

**Software:** Hai-Yan Deng, Lin-Li Pu, Rong Gou.

**Writing – original draft:** Deng-Rong Zhou, Hai-Yan Deng, Lin-Li Pu, Shao-Lan Lin, Rong Gou.

**Writing – review & editing:** Feng-Ling Wang.
